# Differential miRNA expression in the three-spined stickleback, response to environmental changes

**DOI:** 10.1038/s41598-017-18128-w

**Published:** 2017-12-22

**Authors:** S. M. Rastorguev, A. V. Nedoluzhko, N. M. Gruzdeva, E. S. Boulygina, F. S. Sharko, A. S. Ibragimova, S. V. Tsygankova, A. V. Artemov, K. G. Skryabin, E. B. Prokhortchouk

**Affiliations:** 1 National Research Center “Kurchatov Institute”, Kurchatov sq. 1, 123182 Moscow, Russia; 2 Institute of Bioengineering, Research Center of Biotechnology of the Russian Academy of Sciences. 33, bld. 2 Leninsky Ave, Moscow, 119071 Russia; 3 Lomonosov Moscow State University, Faculty of Biology, Leninskie Gory 1-12, 119991 Moscow, Russia

## Abstract

miRNAs play important role in the various physiological and evolutionary processes, however, there is no data allowing comparison of evolutionary differences between various ecotypes adapted to different environmental conditions and specimen demonstrating immediate physiological response to the environmental changes. We compared miRNA expression profiles between marine and freshwater stickleback populations of the three-spined stickleback to identify the evolutionary differences. To study the immediate physiological response to foreign environment, we explored the changes induced by transfer of marine sticklebacks into freshwater environment and vice versa. Comparative analysis of changes in miRNA expression suggested that they are driven by three independent factors: (1) non-specific changes in miRNA expression under different environmental conditions; (2) specific response to freshwater conditions in the marine stickleback ecotype; (3) specific response to extreme osmotic conditions for both marine and freshwater ecotypes during the contact with non-native environment. Gene Ontology enrichment analysis of differential expressed miRNA targets supports our current hypothesis.

## Introduction

MicroRNAs (miRNAs) are small (approximately 22 nucleotides in length) non-coding RNAs involved in post-translational regulation of gene expression. MiRNA genes contain palindromic sequences and are transcribed by RNA polymerase II or III into precursors that are processed to the mature miRNAs by enzyme complex, including Drosha and Dicer enzymes. The mature miRNAs become then incorporated into the RNA-induced silencing complex (RISC) and participate in the degradation or transcriptional silencing of messenger RNAs (mRNAs)^1,2^. It has been shown that this type of regulation is widespread in the animal kingdom^3^ and in plants^4^, but the processing of miRNAs in plants has distinctive features^5^.

miRNAs are widespread in fish and play an important role in embryonic development, morphology, organogenesis, and other processes^6–13^.

It is also known that miRNAs play a role in osmoregulation in fish: in zebrafish (*Danio rerio*), miRNAs of miR-8 family (miR-200a, miR-200b, miR-200c, miR-141, and miR-429) are expressed in ionocytes, specialized epithelial cells involved in ion homeostasis maintenance, and control the ion transport by modulating Nherf1 gene expression;disruption of miR-8 family member function results in failure to respond to osmotic stress and traffic transmembrane glycoproteins^14^.

It was demonstrated that miR-30c is an important regulator of osmotic balance in Nile tilapia (*Oreochromis niloticus*); loss of function of this miRNA expressed in kidney resulted in disruption of the response to osmotic stress^15^. Another member of miR-8 family in Nile tilapia - miR-429 - directly regulates the transcription factor of osmotic regulation 1 (OSTF1), loss of miR-429 functions substantially increases OSTF1 level and leads to changes in the ionic concentration and osmotic stress^16^.

Three-spined stickleback (*Gasterosteus aculeatus*) is a convenient model system to study the process of adaptive speciation in changing habitats^17^ as its marine form repeatedly colonized freshwater habitats alongshore of the Northern hemisphere. Native marine three-spined stickleback population uses freshwater streams and lakes for spawning. However, isolation in new freshwater habitats results in the development of freshwater resident population that eventually changes the morphotype and acquires other features that allow surviving in freshwater habitats. These features make the three-spined stickleback a useful model for the adaptive evolution studies.

There are several studies on genome-wide changes in the process of adaptive speciation in the three-spined stickleback^18–20^ that identified genomic “islands of divergences” - loci with high concentration of “freshwater” polymorphisms.

miRNA investigations in the three-spined stickleback were conducted both by miRNA sequencing of brain tissues^21^, where it was shown that miRNA profiles of the Japanese three-spined sticklebacks vary according their origin, and by the computational approach searching for miRNAs in the genome of the stickleback^22^. In addition, the miRNA sequencing of marine and freshwater stickleback gills, SNP search and comparison with “islands of divergence” was performed to identify miRNAs involved in freshwater adaptation^23^. However, miRNA differential expression analysis in freshwater and marine forms has not been published yet.

Here we aimed at identification of miRNAs role in sticklebacks’ freshwater adaptation. We have performed RNA sequencing analysis of miRNA isolated from the gills of three-spined sticklebacks from marine and freshwater populations, as well as sticklebacks that were kept for several days in an unusual environment (marine stickleback was kept in a fresh water and freshwater stickleback - in a marine water). We identified expression profile in native controls and between each control and case set. While designing the experiments, we planned to analyze “immediate response” miRNA expression changes, when transferring samples of marine sticklebacks in freshwater, in comparison with “evolutionary” miRNA expression changes between the marine and freshwater samples. In other words, we questioned whether the miRNA regulation mechanisms of evolutionary adaptation to fresh water correspond to physiological (instant) mechanisms when samples are placed in the fresh water. Based on our results, here we proposed the hypothesis of three separate trends that modulate the miRNA expression: 1) non-specific changes under different environmental conditions; 2) specific response to freshwater conditions in the marine sticklebacks; 3) specific response to extreme osmotic conditions for both ecotypes.

## Materials and Methods

### Sampling and experimental design

Ten marine three-spined stickleback specimens were collected in the White Sea near the Pertsov White Sea Biological Station of Lomonosov Moscow State University (WSBS MSU) (Murmanskaya oblast, Russia). Ten freshwater three-spined stickleback specimens were collected in Mashinnoe Lake near the village Chkalovsky (Republic of Karelia, Russia). The lake age since desalination, inferred from its current elevations above the sea level, is 700 years^24^, and it is situated far enough from the White Sea ensuring freshwater origin of the population.

The marine and freshwater sticklebacks were markedly different from each other in a number of morphological characteristics^25^ that significantly reduced the chance of error (mixing up the intermediate or freshwater forms of stickleback, which were accidentally brought into the sea). To synchronize the physiological status of the samples, only males in breeding plumage were collected. Collected samples were transferred in water tanks; for four days, half of the samples in each group was kept in native water environment, whereas the other half - in modified water environment (freshwater samples in seawater and vice versa). Control samples were kept to compensate captivity stress of the case samples. This experimental design allowed us not only to identify differences in miRNA expression between marine and freshwater form of the three-spined stickleback, but also to study the change of expression profile that occurs during the transition to the unusual environment analyzing evolutionary and physiological mechanisms of freshwater adaptation by miRNA regulation. Gill tissues of all twenty samples were isolated and fixed in IntactRNA® reagent (Evrogene, Russia). Then gills were isolated from 20 samples (5 samples for each experiment and control), the gills were chosen as target tissues, as they, along with the kidneys, serve as an important organ of osmotic regulation, and we expected the most important for the water-salt balance miRNAs to be expressed in gills. Moreover, compared to kidneys, gills are more convenient tissues to be identified and isolated during experiments helping to reduce the experimental error rate. miRNA libraries were made as described in material and methods and sequenced on Illumina instruments.

This work was carried out in accordance with relevant guidelines and regulations and was approved by ethical committee of Research Center of Biotechnology RAS, Moscow, Russia.

### miRNA extraction from gill tissues

TRIzol® Reagent was used for total RNA extraction from *G.aculeatus* gill tissues using standard protocol (Invitrogen, USA). The RNA concentration in the samples was measured using a BioAnalyzer 2100 (RNA 6000 Nano Kit) (Agilent, USA).

### cDNA library construction

Twenty multiplexed Illumina miRNA libraries were constructed using the NEBNext Small RNA Library Prep Set for Illumina (NEB, UK), following manufacturer’s instructions (five libraries from each control and case samples of the three-spined sticklebacks). Concentration and quality of constructed miRNA libraries were evaluated using Agilent Bioanalyzer 2100 (Agilent Techonologies, USA). *G. aculeatus* cDNA libraries were used for sequencing using Illumina GAIIx platform with 36 bp long reads.

### mRNA sequencing and RNA-seq analysis

Extraction and sequencing of mRNA and RNA-seq analysis were described in^26^.

Four fish from marine population and four fish from freshwater population were taken for transcriptome analysis. Gills were isolated and fixed with IntactRNA® reagent (Evrogen).

Total RNA was extracted from the samples with Trisol reagent according to the manufacturers instructions (Invitrogen). Quality was checked with the BioAnalyser and RNA 6000 Nano Kit (Agilent). PolyA RNA was purified with Dynabeads® mRNA Purification Kit (Ambion). An Illumina library was made from polyA RNA with NEBNext® mRNA Library Prep Reagent Set (NEB) according to the manual. Paired-end sequencing was performed on HiSeq. 1500 with 2 × 75 bp read length. Approximately 25 million reads were generated for each sample.

Reads were mapped to gasAcu1 genome with tophat2 software (version 2.1.0)^27^. Number of RNA-seq reads, number of unmapped reads and mapping efficiency is summarized in Supplementary table S6. Gene models of non-overlapping exonic fragments were taken from ENSEMBL 54 database. For each exonic fragment total coverage by mapped reads in each sample was calculated with bedtools multicov tool (version 2.17.0). Total gene coverage was calculated as a sum of coverages of all non-overlapping exonic fragments of a gene.

### Sequence analysis and miRNA identification

miRNA gene positions were used as described previously^23^. The reads of the sequenced libraries were filtered by quality (phred > 20) and size (reads with <17 bp were eliminated); the library adapters were eliminated using Cutadapt software (version 1.8.3^28^), adapter sequences used were - ATCTCGTATGCCGTCTTCTGCT, AGATCGGAAGAGCACACG, TACAGTCCGACGATC, AGACGTGTGCTCTTCCGATCT, GTTCAGAGTTCTACAGTCCGACGATC.

miRNA genes prediction was conducted using miRDeep2 version 2.0.0.5 program^29^, which allows to determine palindromic sequences around homologous sites and compare the results with the known miRNA genes in the databases.

The three-spined stickleback genome from the Ensembl database (BROAD S1, Feb 2006, assembly 61; http://www.ensembl.org)^30^ was used as a reference genome. GAmiRdb database was used as a resource for searching for miRNAs and their targets in the three-spined stickleback^22^.

The mapping of Illumina reads was conducted using *bowtie2* tool (version 2.2.3^31^) and bwa*_sam_converter.pl* script from the miRDeep2 package (to convert *.*sam* to *.*arf*). For bowtie2 the “–very-sensitive” parameter set was used.

The BED-files (*.bed), containing the coordinates of miRNAs, were generated for genes that were predicted by miRDeep2 package during Illumina reads analysis. These genes were combined into a single set using *mergeBed* utility from the *Bedtools* software package (version 2.17.0^32^).

### Differential expression analysis of the three-spined stickleback microRNA genes

To determine the differentially expressed miRNA genes, Illumina reads were mapped on the genome of the three-spined stickleback from Ensembl database using *bowtie2* program with SAM files (*.sam) as output. SAM files were compressed, sorted and indexed by *SAMtools* software packages (version 0.1.19-44428 cd)^33,34^. Each miRNA gene coverage was calculated by *coverageBed* utility of the *bedtools* software package using bed file with miRNA gene coordinates and BAM files (*.bam) as input. Exact command line was: “bamToBed -i infile.bed | coverageBed -a stdin -b miRNA_gene_position.bed > output.coverage”. Each gene coverage data were used for gene activity table taking by custom perl script. The script takes coverage files from coverageBED of bedtools output (which is single file for each library) and combines all library and all loci data in common table. The difference in gene expression was calculated by edgeR package (version 3.14.0^35^) of R software environment for statistical computing (http://www.r-progect.org).

### Correlation analysis

For correlation analysis, we estimated DE (difference expression) statistics between each pairs of experimental groups with edgeR package. As necessary step for DE analysis, the logFC (logarithm of expression fold change) should been estimated, and that measure we used for assess correlations between each group transition. Correlations were calculated with cor.test() function of R statistics environment. The logFC metric was estimated for marine-freshwater (SfSw-FfFw) groups, marine-marine freshwater kept (SfSw-SfFw) and freshwater-freshwater seawater kept (FfFw-FfSw). Correlations were estimated between: logFC(SfSw-FfFw) vs logFC(SfSw-SfFw) - concordance between evolutional and physiological miRNA expression change as response on fresh water; -logFC(SfSw-FfFw) vs logFC(FfFw-FfSw) - concordance between evolutional and physiological miRNA expression change as response on sea water; logFC(FfFw-FfSw) vs logFC(SfSw-SfFw) - physiological miRNA expression change as response on change water enviroment.

### miRNA target gene prediction

The prediction of target genes for the miRNAs was performed using miranda software^36^, which uses the local homology between the mature miRNA nucleotide sequence and proposed target gene sequence. As the target sequences, we used cDNA sequences of each three-spined stickleback gene that were obtained by BioMart Ensembl service.

### Gene Ontology (GO) enrichment analysis of miRNA targets

After the target gene prediction for individual miRNAs, we converted stickleback Ensembl IDs into IDs of *Danio rerio* orthologs using BioMart Ensembl service and performed GO enrichment for the predicted targets using Gorilla web service^37^. This conversion is necessary because the service does not use the three-spined stickleback genome as a reference for searching for enriched GO terms. The single ranked test was conducted.

For the miRNA target enrichment analysis, we used bioconductor^38^, package miRNApath (version 1.34.0^39^). We specifically considered only potential target genes which had detectable transcription (mean normalized transcription level between samples had to be at least 10 reads per gene). To study miRNA - target relationships, we used miranda software, as described above, and the correspondence of each gene to specific GO categories was defined using BioMart service of Ensembl genome database.

## Results

### RNA sequencing analysis

Quantities of obtained and mapped Illumina reads for each library are present in Supplementary Table S5.

The sample FfSw4 was removed from further analysis, due to it’s low read coverage. At first, these data were used to find additional miRNA genes using miRDeep2 program, as was done and described earlier^23^. In addition to 595 previously defined miRNAs, 244 new miRNAs were identified as expressed in the three-spined stickleback gills – in total, resulting in 839 miRNA genes. Generated sequencing reads were mapped to the genome of the three-spined stickleback and intersected with the coordinates of the identified miRNA genes. A total of 5,505,821 sequencing reads were mapped on the miRNA genes for all 20 libraries.

### miRNA differential expression

**Figure 1:**
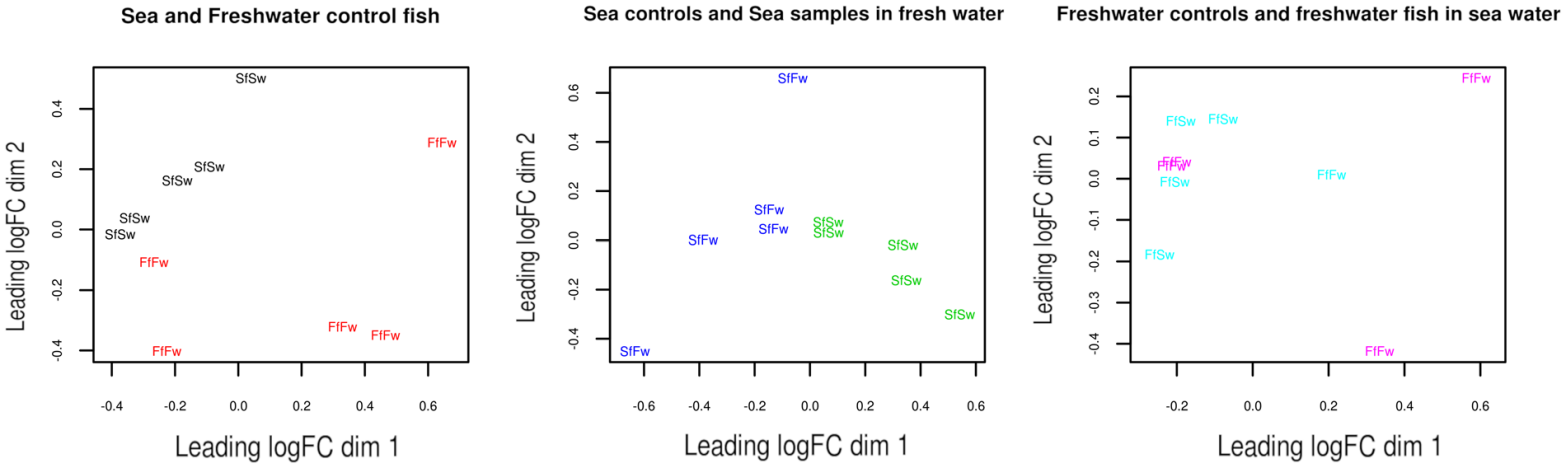
MDS scatterplots of experimental samples based on differential expression data: (**A**) MDS for control marine and freshwater samples; (**B**) MDS for control marine and fresh water-kept marine samples; (**C**) MDS for control freshwater and seawater-kept fresh water samples).

We compared miRNA expression between marine and freshwater sticklebacks and discovered that 6 out of 839 miRNAs were differentially expressed between these conditions. Meanwhile, a comparison of marine sticklebacks kept in marine and freshwater environments yielded 18 differentially expressed genes. Different number of discovered genes can be explained by the fact that miRNA expression in freshwater samples has a high dispersion and comparison with freshwater samples greatly decreases the statistical significance of differences for other compared samples. The multidimensional scaling (MDS) plot shown in Fig. 1 demonstrates differential miRNA gene expression between control marine and freshwater samples (Fig. 1A); between control marine and freshwater-kept marine samples (Fig. 1B); and between control freshwater and seawater-kept freshwater samples (Fig. 1C). Our data show that freshwater samples are wide scattered on the plot, suggesting that their miRNA expression is more heterogeneous compared to miRNA expression in marine samples.

Due to such high heterogeneity, a statistically significant difference in miRNA expression between the control freshwater group and seawater-kept freshwater samples was found only for one miRNA – miR184. Moreover, this miRNA was also shown the most significantly differentially expressed between marine and freshwater controls. The expression of this miRNA was found increased in the freshwater samples and decreased in the marine samples. In addition, when freshwater sticklebacks were placed into marine water, the expression of the miRNA in these freshwater samples became significantly decreased (up to the level of seawater samples). Difference expression data are present in Supplementary Tables 2, 3 and 4.

However, after four days of the opposite experiment when marine sticklebacks were placed in fresh water the expression of this miRNA did not increase to a “freshwater” level. This miRNA belongs to a conservative family of miR-184 and is located in the middle of the second chromosome of the three-spined stickleback, away from freshwater “islands of divergence”^20^ and more than 15 thousands of nucleotides away from the nearest protein-coding gene. According to published data in zebrafish, this miRNA is actively expressed in lens, hatching gland and epidermis^11^, In mammals, mature miR-184 is particularly enriched in nervous tissue, testis and corneal epithelium^40,41^ and in Drosophila, mir-184 is expressed ubiquitously in all tissues from embryonic to adult stage, and its expression pattern dynamically changes during the development of the embryo, especially in the central nervous system^42^.

In the study of differential gene expression, mir-8 family was shown to play a role in osmoregulation in zebrafish and tilapia^14,15^. Here, we found that mir-200a (a member of mir-8 family) was involved in freshwater adaptation in the three-spined stickleback When placing marine fish into fresh water, the expression of mir-200a significantly increased almost twofold (False Discovery Rate = 0.027), while the expression levels of this miRNA in marine and freshwater controls were similar and, when freshwater samples were placed into the seawater, the level of the mir-200a remained the same.

### Correlation analysis

**Figure 2:**
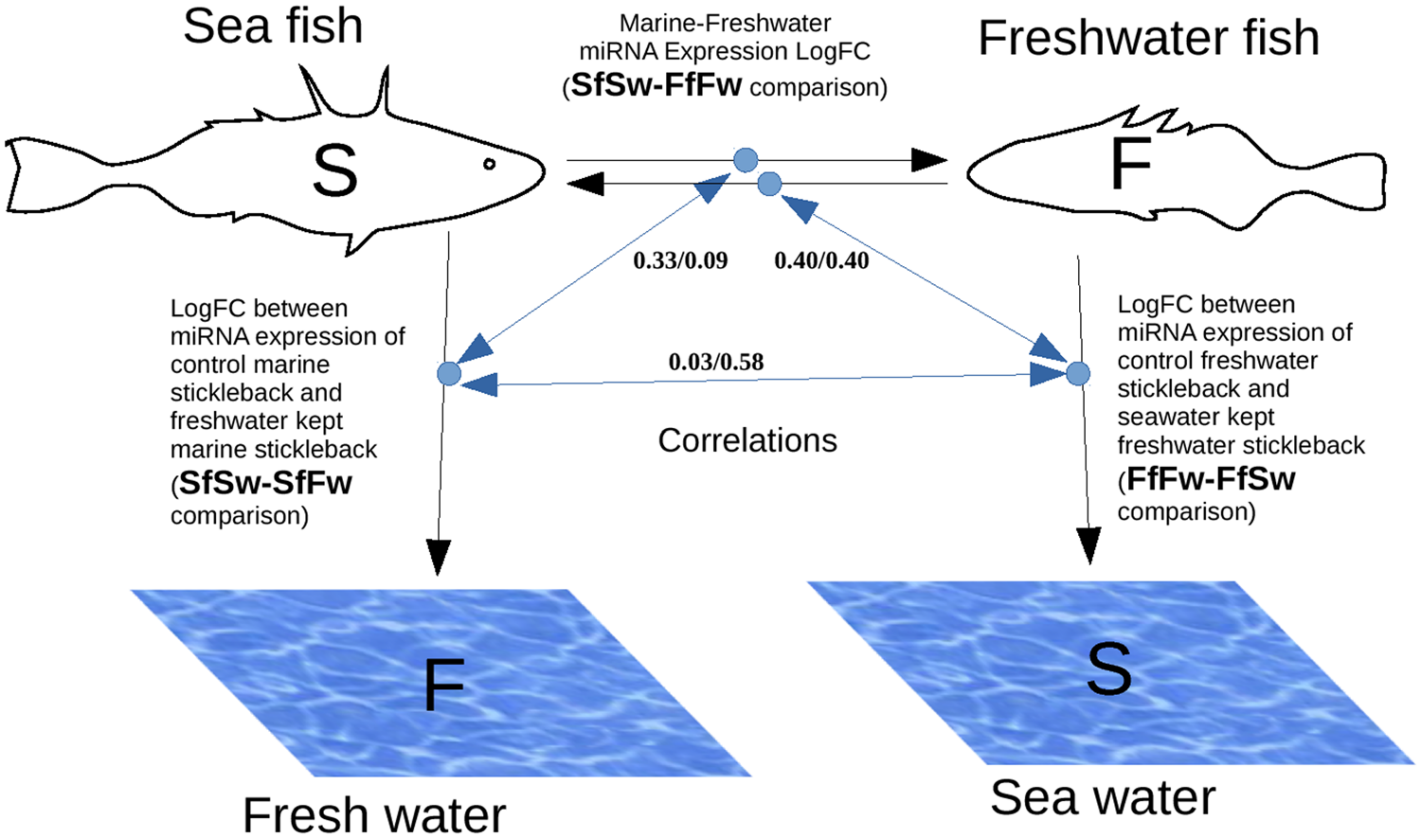
Experiment outline. Correlation values between inter-sample expression fold change. The values show correlations for full dataset and highly expressed miRNA (see explanations in the text).

To further study the changes of miRNA expression in different sample groups, correlation analysis of expression changes between the control and experimental samples was performed. Correlations were found between the logarithmic fold changes of gene expression in each experiment as shown in Fig. 2.

Significant correlation was observed between miRNA expression fold changes in samples transferred to foreign environment (SfSw → SfFw and FfFw → FfSw comparison) and changes in control samples (SfSw and FfFw comparison) (Fig. 2).

no correlations between miRNAs expression changes after transferring to unusual environment (SfSw → SfFw comparision and FfFw → FfSw comparision) were discovered.

Pearson test showed a statistically significant correlation value of −0.33 between SfSw ↔ FfFw and SfSw ↔ SfFw (*p-value* < 2.2 e-16). Moreover, significant correlation value of −0.4 was observed between expression changes for SfSw ↔ FfFw and FfFw ↔ FfSw (*p-value* < 2.2 e-16). We also observed no significant correlation between for SfSw ↔ SfFw and FfFw ↔ FfSw (0.03 with *p-value* = 0.3497).

**Figure 3:**
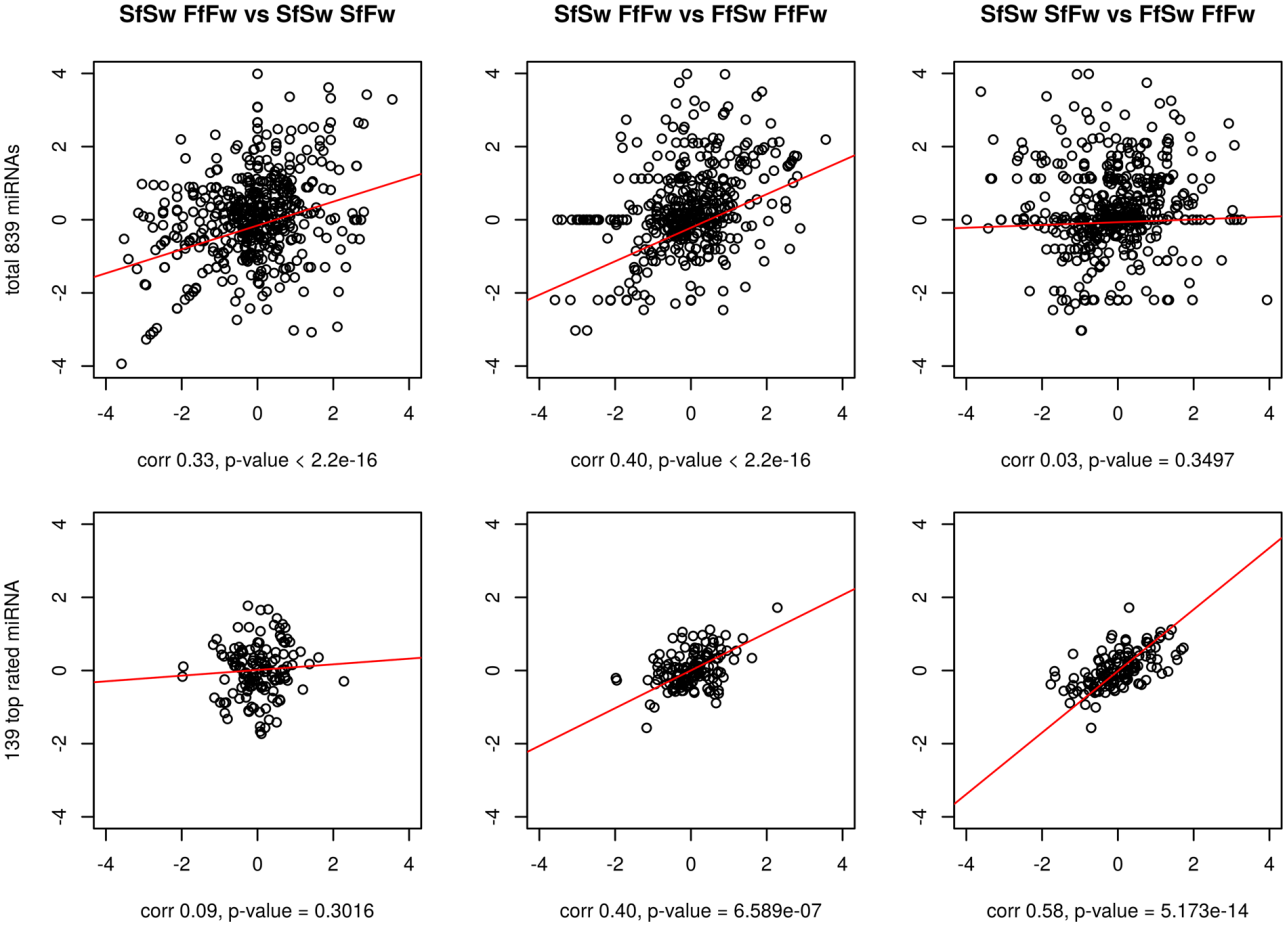
Scatterplots of normalized miRNA expression between different experimental groups. Group labelling as in Fig. 2. (**A**) Correlation of all 839 miRNAs; (**B**) Correlation of 139 top expressed miRNAs.

The described effects were observed only when all generated data were used (even miRNAs with moderate and low expression level and possibly without an effect on another gene expression). When we used only highly expressed microRNA genes, the statistic pattern changed. The correlation between expression changes in SfSw ↔ FfFw and SfSw ↔ SfFw disappeared but significant positive correlation between SfSw ↔ SfFw and FfFw ↔ FfSw was found; the correlation between SfSw-FfFw and FfFw ↔ FfSw did not change (Fig. 3).

For example, for the genes which were covered by more than 1,000 reads in each cDNA-library (in total, 139 miRNA genes), the correlation between SfSw-FfFw and SfSw-SfFw appeared to be 0.09 (*p-value* = 0.3016) and the correlation btween SfSw ↔ SfFw and FfFw ↔ FfSw was 0.58 (*p-value* = 5.173 e-14).

### Target prediction for differentially expressed miRNAs

First, we tried to identify the target genes for miR-184, since it is the only differentially expressed miRNA that was found in two experimental groups - differentiation between marine and freshwater controls and between freshwater control samples and “seawater-kept” freshwater samples.

The analysis of targeted genes for miR-184 showed that in human, according to the database microRNA.org^43^, this miRNA regulates more than 600 genes (considering only the results of the high mirSVR values), and two orthologs of these genes in the three-spined stickleback are located in “islands of divergence”^20^ - a transcription factor stat5a and histone methyltransferase ezh1. Both genes encode gene regulators of broad spectrum activity.

miRNA target prediction can also be done by bioinformatics methods using the local homology of the mature miRNA sequence and target gene sequence. Although such methods are known by “high positive rates”^44^, they are rather widely used in miRNA research. We used miranda software package for such predictions^36^ and showed that 293 genes of *Gasterosteus sticklebacks* from Ensembl database have local homology to mir-184 in the 3′ noncoding region and 4359 genes - in other cDNA sequences. GO analysis of the potential targets (we converted stickleback genes into their orthologs in *D. rerio*) did not find any enriched GO category.

Modern methods of data generation and analysis provide a wide range of options to identify complicated interrelations between different types of data. There are a number of methods and software packages to detect enriched GO categories and pathways among miRNA target. We used one of these approaches implemented in bioconductor package miRNApath^39^; this package uses differential miRNA expression data and miRNA-target interactions and looks for pathways and GO categories that are enriched in a pool of targeted genes. This way, one can try to predict what biological processes are affected by miRNA changes. The package is designed to preserve the additive effects of miRNAs on genes that should improve the prediction accuracy.

**Figure 4:**
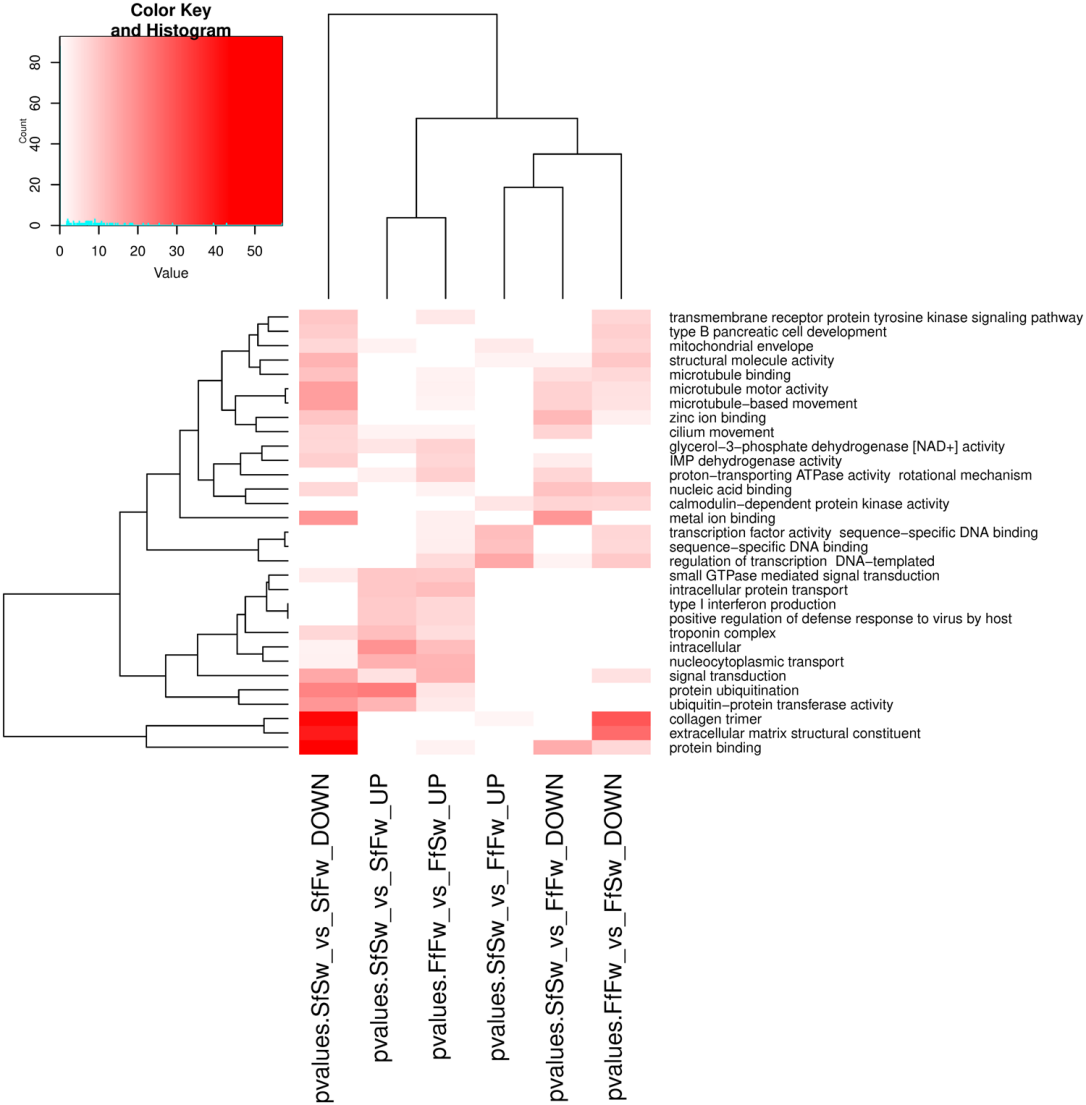
GO enrichment test for putative target genes of differentially expressed miRNAs. Vertically marked with the enriched Gene Ontology terms, horizontally – comparison group of sticklebacks. Target analysis of UP and DOWN expressed miRNA was performed separately.

Results of GO enrichment for target genes of differentially expressed miRNAs are shown in Fig. 4. The separate analysis was done for the UP and DOWN expressed miRNA genes in each of the three experimental groups:

1. Differentially expressed miRNA genes between marine and freshwater controls SfSw-FfFw (UP/DOWN).
2. Differentially expressed miRNA genes between marine controls and “fresh water-kept” marine samples SfSw-SfFw (UP/DOWN)
3. Differentially expressed miRNA genes between freshwater controls and seawater-kept freshwater samples FfFw-FfSw (UP/DOWN).

It is shown that when we cluster the experimental groups by mutual GO enrichment of targets for differential expressed miRNA, the closest to each other groups are “seawater-kept” freshwater sticklebacks and “fresh water-kept” marine sticklebacks. The same groups (regardless of UP/DOWN expression) correlate to each other by miRNA gene expression (Fig. 3). For DOWN expressed miRNA, these same groups are the ones that are most different from others and they are also most GO enriched by different categories. These categories can be interpreted as those, for which miRNA regulation is alleviated by unusual environment.

## Discussion

### Correlation analysis

According to a previous study, there are no significant nucleotide substitutions in functional miRNA sequences (mature or star sequences) of genes that are active in the three-spined stickleback gills^23^. To further determine the role of miRNAs in the adaptive speciation, we analyzed the differential expression of miRNA genes in marine and freshwater sticklebacks with regards to long-term mechanisms and short-term response.

The main objective of this work was to determine whether the long-term evolutionary mechanisms of adaptation to the fresh water by the miRNA expression shift coincide with the rapid physiological response to changes in salinity. As an indicator of evolutionary mechanisms, we used the difference in miRNA expression between native marine and freshwater samples, suggesting that it was formed during many generations as a result of adaptation of originally marine sticklebacks to freshwater environment. This difference in expression was compared with the difference that arose when transferring control samples into the non-native environment - marine fish into fresh water (SfFw) and freshwater fish into the seawater (FfSw).

As mentioned above, when transferring marine three-spined sticklebacks into fresh water, 18 microRNA genes significantly changed their expression, but none of these genes coincide with differentially expressed genes of marine and freshwater controls. Since the simple comparison of differentially expressed genes did not work, we found another way to compare the experimental results: the correlations of logarithmic fold-change of gene expression were chosen as the indicators of miRNA expression changes under environmental influence.

Based on the initial assumptions, expression changes logarithm (logFC) between control marine and freshwater samples should be correlated to that between marine control samples and freshwater-kept marine samples - this would serve as evidence that the physiological response to changes in salinity coincides with an evolutionary response. Thus, during long evolution period, freshwater forms establish and sustain such miRNA expression changes that occur in marine sticklebacks placed in freshwater during the physiological response to the fresh water. On one side, we can see such an effect: if we look at the correlation based on the full dataset, we can see that these changes significantly correlated. However, it does not explain why genes that are differentially expressed in control marine and freshwater samples do not match with miRNA genes that are differentially expressed when marine samples are placed into fresh water. Moreover, this correlation disappears if we exclude from the analysis the weakly expressed genes.

The fact that we do not see correlation with “highly expressed miRNA genes” set could mean that miRNA regulation of the quick physiological response to salinity does not coincide with the slow evolutionary response. In other words, the transfer of marine samples into fresh water turns on different miRNA regulation mechanisms compared to those that freshwater populations form during many generations to adapt to freshwater environment.

As a result of the opposite transfer (freshwater samples to seawater), we see statistically significant correlation of miRNA expression changes that reflects the situation with mir-184, which is differentially expressed both between freshwater and marine controls and during transfer of freshwater samples to salt water.

Further on, when transferring marine samples into fresh water and freshwater samples in see water, we expected that we should get negative correlation, since those are opposite adaptation conditions and physiological processes should also be opposed directed. However, with the set of highly expressed miRNAs (considering just the functional effect), we found out a significant positive correlation.

As a result of the analysis, we propose, as we believe, the most consistent hypothesis explaining the observed effects. From our point of view, this situation can be explained by combination of three separate trends, which modulate the miRNA expression in general:

1. First, as a result of either direct action of the environment on the miRNA expression or mediated by some non-specific regulation mechanisms, the expression of all or most of the miRNA genes is shifted in freshwater. Such non-specific effect is most noticeable on the weakly expressed miRNAs, so when we take into account all miRNAs, we obtain significant correlations between controls and each experimental group. However, given the fact that the regulation of miRNAs, particularly weakly expressed ones, can only slightly adjust the activity of other genes, this trend is unlikely to have a significant impact on the overall expression. So, such correlation is likely nonfunctional, and is caused by environmental exposure. Highly expressed miRNA genes, such as mir-184, also belong to this group that reveals differentiation of expression between the controls and during transfer freshwater samples to seawater. When we exclude the low-expressed miRNA genes from the analysis, the other effects are revealed:
2. When transferring marine samples into fresh water, there is a specific miRNA response to freshwater conditions, which obviously is a functional, as it appeared only in highly expressed miRNA genes and does not correlate with the effect №1. In this case, we do not see the opposite effect, when moving freshwater samples into seawater (there is just a correlation with the control), and this situation is easily explained by the fact that the native marine sticklebacks go into fresh water for spawning, and they need a such sort of mechanisms to better prepare themselves for the change of environment, meanwhile, freshwater sticklebacks, at least in our study population, reside in fresh water and do not contact with salt water at all, so they do not need this kind of arrangements. The miRNA of this type is mir-200a, which increases its expression when marine samples are placed in fresh water; it belongs to the mir-8 family, which has been shown to play an important role in adaptation to salinity change in zebrafish and tilapia^14,15^.
3. The comparison between the two experiments — marine stickleback in fresh water and freshwater stickleback in seawater, shows that for highly expressed miRNA genes there is reliable positive correlation between miRNA expressions. The fact that expression of some of the miRNAs varies in a similar way, both when fish is placed into sea or fresh water, suggests that in this case, the factor influencing the miRNAs expression is not the water salinity, but rather physiological stress when organisms is adapting to unusual environment - one more miRNA expression changes’ trend we have identified.

Thus, according to our hypothesis, we found three types of responses: 1. non-specific miRNA response, which reflects the difference between the miRNA expression in marine and freshwater controls, and between freshwater controls and freshwater experimental samples in seawater; 2. specific response to fresh water from marine samples; 3. specific stress response as a result of exposure to unusual environment for both experiments (marine stickleback in fresh water and freshwater stickleback in seawater).

The idea of non-specific miRNA response under the action of the environment comes from the fact that this effect is seen mainly on weakly expressed miRNAs, however, some highly expressed miRNAs can also have shifted expression in different salt conditions, so it is possible that this is also a specific trend for better adaptation to different conditions of environmental salinity using miRNA regulation.

### Target analysis

Current bioinformatics miRNA target prediction methods such as existing algorithms for the miRNA target prediction by the local homology of mature miRNA sequences to 3′ non-coding regions of genes or cDNA sequences are shown to have a very high false positive rate^44^. However, if these false predicted targets are distributed randomly among GO categories, one can expect more or less realistic results for the GO enrichment analysis for miRNA target, and, according to enrichment algorithm, GO categories should not be enriched randomly, although false positive results can “mask” the enrichment of some under-represented GO categories.

The results of the above-mentioned analysis, conducted for our differentially expressed miRNA genes, are shown in Fig. 4. The obtained results look meaningful with regards to our suggested hypothesis. First, when we clustered experimental groups of samples by common enriched GO categories, the closest groups, which share common cluster, were the groups of sticklebacks placed in non-native environment, based on the targets of UP expressed miRNAs (two left columns on heatmap, Fig. 4), and the same groups (FfFw-FfSw ~ SfSw-SfFw) revealed the most highly correlated miRNA expression changes for highly expressed genes. Further on, GO categories commonly enriched between the two groups included; ≪type I interferon production≫ and ≪positive regulation of defense response to virus by host≫ that can be referred to the categories associated with response to stress. The categories related to muscular activity (≪troponin complex≫) can also be associated with response to stress. As mentioned above, according to our hypothesis, when changing the environment, specific miRNAs are activated and that are involved in the response to the environmental stress, and the data of GO enrichment for miRNA targets correlate well with our assumption.

The categories which show different enrichment between FfFw-FfSw and SfSw-SfFw comparisons included ≪proton–transporting ATPase activity≫, ≪integral component of plasma membrane≫, ≪calcium ion homeostasis≫. These categories can be associated with a specific response to the changes of environmental salinity.

As shown on the heatmap (Fig. 4), the control groups (GO enrichment among targets of differentiated miRNA between native marine and freshwater samples for the UP and DOWN expressed miRNAs) form a single cluster. This means that both UP and DOWN expressed miRNAs from the control group regulate genes from more or less the same GO categories that indirectly confirms one of the points of our hypothesis that the difference between marine and freshwater controls is not functional, but depends on non-specific environmental influences.

### Data Accessibility

A list of miRNAs, discovered in the investigation, with gene sequences and genomic positions, is available in Supplementary Table 1. Lists of differential expressed miRNAs with expression statistics are available in Sapplemetntary Tables 2–4: for marine – freshwater controls, for marine controls and freshwater-kept marine samples and for freshwater control and seawater-kept freshwater samples accordingly. The row sequencing data available in NCBI SRA database under BioProject ID PRJNA420035.

## Electronic supplementary material

Supplementary Dataset 1

Supplementary Dataset 2

Supplementary Dataset 3

Supplementary Dataset 4

Supplementary Dataset 5

Supplementary Dataset 6
